# The influence of team reflexivity on employee’s feedback-seeking behavior: a multi-level perspective

**DOI:** 10.3389/fpsyg.2024.1512075

**Published:** 2025-02-07

**Authors:** Mengli Liu, Bing Liu, Qihui Sun, Huijuan Wang, Yixin Zhang

**Affiliations:** School of Management, Shandong University, Jinan, China

**Keywords:** team reflexivity, transactive memory system (TMS), feedback-seeking behavior, shared mental models (SMMs), transactive memory system theory, multi-level analysis, team dynamics, organizational behavior

## Abstract

The dynamism and uncertainty of the work environment increasingly emphasize the importance of employee’s feedback-seeking behavior. Based on transactive memory system theory, the current study explores the multi-level effect of team reflexivity on team member’s feedback-seeking behavior. Survey data collected from 197 participants in 56 teams in China showed that team reflexivity had a positive indirect effect on team member’s feedback-seeking behavior, and team transactive memory system (TMS) mediated such effect. In addition, results also indicated that team shared mental models (SMMs) moderated the effect of team reflexivity on TMS. The research findings can help organizations recognize the importance of team reflexivity, TMS, and SMMs in promoting employees’ feedback-seeking behavior. Based on this understanding, organizations can better formulate strategies to leverage team reflexivity, TMS, and SMMs to enhance team member’s feedback-seeking behavior, which will also be beneficial for the sustainable development of the organization.

## 1 Introduction

With the growing dynamism of the environment, team reflexivity becomes especially important as it has been proved to be a key approach for organizations to monitor and react to environmental changes (Schippers et al., 2014; Widmer et al., 2009). Team reflexivity refers to the extent to which team members overtly reflect upon and communicate about the team’s objectives, strategies, and processes, and adapt themselves to current or anticipated dynamic environments (West, 1996, 2000). Previous research has mainly focused on exploring the positive effects of team reflexivity on teams, including team performance (Yang et al., 2020; Wu et al., 2019; Schmutz et al., 2018; Schippers et al., 2013), team innovation (Leblanc et al., 2022; Chen et al., 2016; Schippers et al., 2012; Tjosvold et al., 2004), team effectiveness (Widmer et al., 2009), and team ambidexterity (Li et al., 2018). Only a few studies have examined the impact of team reflexivity on team members, such as employee’s psychological well-being (Chen et al., 2018) and employee’s innovative behavior (Wang et al., 2021; Wang et al., 2022).

However, the influence of team reflexivity on team member’s feedback-seeking behavior, which is defined as “conscious devotion of effort toward determining the correctness and adequacy of behaviors for attaining valued end states” (Ashford, 1986, p. 466) has rarely been noted in the dynamic environment. Notably, feedback-seeking behavior is encouraged by prior scholars and practitioners, because positive solicitation of feedback is related to better performance, effectiveness, and creativity of individuals because feedback-seeking behavior provide essential information that allows them to improve (Ashford and Tsui, 1991; De Stobbeleir et al., 2011; Gong et al., 2017; Sherf and Morrison, 2020). Particularly in the growing dynamism of the environment, information tends to be more uncertain and complex and is often not volunteered by others,which increases the value of feedback seeking (Bennett and Lemoine; 2014; Sherf and Morrison, 2020; Ashford, 1986). As a result, it becomes especially significant for employees to seek feedback from others when facing the uncertainty of the work environment. What’s more, team reflexivity focuses on team level to explain the adaptation of working methods to changing environments (West, 1996). Whereas considering adaptive behavior at the micro level, it is closely related to feedback-seeking behavior in terms of collecting information by individuals to adjust their behavior to the environment (Anseel et al., 2015; Ashford and Cummings, 1983; Bălăceanu et al., 2021). In light of this, feedback-seeking can be viewed as a crucial consequence to evaluate the effectiveness of team reflexivity in practice. Accordingly, scholars believed that team reflexivity may produce a supportive feedback environment by requiring team members to openly discuss appropriate ways to collaborate and to periodically revise objectives and procedures (Sung et al., 2019; Wu et al., 2014), but they did not further reveal the mechanisms of the relationship between team reflexivity and team member’s feedback-seeking behavior. Therefore, this study intends to explore how and when team reflexivity affects team member’s feedback-seeking behavior.

This research draws on the transactive memory system theory (Wegner, 1987) to identify team transactive memory system (TMS) as an important mediator in explaining team reflexivity influencing member’s feedback-seeking behavior. TMS refers to an interdependent cooperation and division system which formed among team members, it is used to acquire, store and use information or knowledge from different fields, which consists of three dimensions: credibility, specialization and coordination (Lewis, 2003; Bachrach and Mullins, 2019). Transactive memory system theory points that interaction and communication among team members can increase the diversity of information and mutual reliance in the team, which is one of the most essential elements for improving TMS (Wegner, 1987; Leo et al., 2022). Thus, with public reflection and communication on the team’s objectives and tasks, team reflexivity may constitute the foundation of the original team TMS structure (Bachrach et al., 2018; Lewis, 2004). Besides, TMS can promote a harmonious and efficient collaborative team atmosphere by enabling members to understand team members’ expertise and increasing their trust about the knowledge and expertise of others (Lewis et al., 2005), which produces a favorable feedback environment characterized by adequate communication and mutual trust. Thus, we believe that TMS mediates the relationship between team reflexivity and team member’s feedback-seeking behavior.

**Figure 1:**
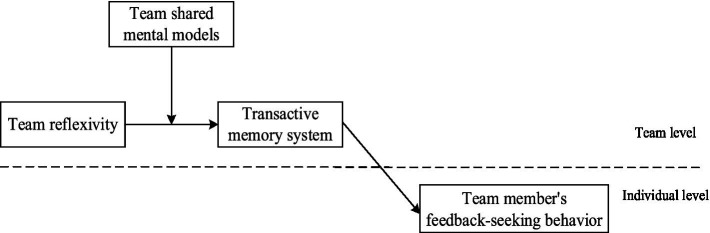
Research model.

We further examine an important boundary condition on the extent to which team reflexivity facilitates TMS and then motivates team member’s feedback-seeking behavior. Given team reflexivity involves the cooperative process of cognitive interaction (Schippers et al., 2013; West, 1996, 2000), the existing of team shared mental models (SMMs), which refers to an organized understanding or mental representation of knowledge shared by team members (Klimoski and Mohammed, 1994), provides a common cognitive basis for team cooperation (Mesmer-Magnus and Dechurch, 2009; Stasser and Titus, 1985). It is crucial to simultaneously consider the cognitive process and characteristic during team cooperation, because the characteristic of high-level shared cognition, in term of SMMs, can help members to reach a consensus on the understanding of the same task, so as to effectively communicate, exchange information as well as coordination (Yang et al., 2008). In addition, according to transactive memory system theory, team members with high-level of shared cognition will be more likely to hold common understandings about their interaction and communication, which is more beneficial to the formation of TMS (Ellis, 2006; He and Hu, 2021). Thus, we believe SMMs will moderate the relationship between team reflexivity and TMS. In particular, when team members hold similar mental models, they are more likely to reach consensus on team knowledge and desired goals during the collective reflection process, and then they can effectively coordinate and utilize their respective expertise to develop TMS of the team. A depiction of our hypothesized model is presented below in Figure 1.

This study attempts to contribute to the existing research in the following ways. First, we extend the individual-level outcomes of team reflexivity by assessing how it affects team member’s feedback-seeking behavior. Previous studies on team reflexivity outcomes are mostly focused on the team level, limiting our knowledge of its impact on team members. Thus, this study extends the literature on team reflexivity by examining how it contributes to team member’s feedback-seeking behavior on the individual level. Second, according to transactive memory system theory, we propose that the relationship between team reflexivity and team member’s feedback-seeking behavior is mediated by TMS. Previous research have already examined the individual psychological and behavioral mechanisms behind the link between team reflexivity on team members (Wang et al., 2021; Wang et al., 2022; Chen et al., 2018), but rarely noticed the impact of team factors. We provided a new theoretical insight on the mechanisms by which team reflexivity affects individual’s behavior from the perspective of the team’s cognitive process (i.e., TMS). Third, our research further contributes to the team reflexivity literature by exploring a boundary condition under which team reflexivity is more or less beneficial for team members. According to transactive memory system theory, we suggest that the relationship between team reflexivity and TMS will be moderated by SMMs.

## 2 Theory and hypotheses

### 2.1 Transactive memory system theory

TMS is defined as an interdependent cooperation and division system which formed among team members, it is used to acquire, store and use information or knowledge from different fields, which is demonstrated to consist of three dimensions, namely: (a) credibility, defined as mutual trust in the skills and knowledge of each team member; (b) specialization, defined as the specific roles that each team member has associated with their skills and knowledge; and (c) coordination, understood as the teams ability to organize and combine different skills and knowledge to be able to work effectively (Lewis, 2003; Lewis et al., 2005). Transactive memory system theory emphasizes the sense of expertise allocation among team members, and encourages members to utilize others’ knowledge storage to complement and enhance their own knowledge (Wegner, 1987; Hollingshead, 1998). Thus, the effectiveness of a TMS relies on cognitive interdependence among members, as they interact with, trust and cooperate with each other.

### 2.2 Team reflexivity and TMS

Based on transactive memory system theory, we anticipate team reflexivity to enable team members to clarify the expertise allocation of the team, create a trusting and collaborative environment, and in turn motivate members to seek feedback from others in the team. Specifically, as a team-regulatory process, team reflexivity involves the process of team members sharing and discussing opinions and ideas about their work (Schippers et al., 2008), facilitating them to develop a more comprehensive understanding and trust of others’ knowledge and expertise (West, 1996). Besides, TMS development requires frequent interaction among members which promotes the integration and utilization of knowledge (Wegner, 1987). Therefore, as a critical and unique interaction process, team reflexivity may serve as a significant factor to improve TMS of the team.

According to transactive memory system theory (Wegner, 1987), we propose that team reflexivity will influence team TMS in three aspects: credibility, specialization and coordination. First, team reflexivity encourages open and honest communication among team members, fostering an environment of trust. As team members engage in reflective discussions, they become more aware of each other’s strengths and weaknesses (Hollingshead and Brandon, 2003), leading to increased credibility in the information shared within the team. This credibility in one another’s knowledge and expertise is a cornerstone of a robust TMS (Brandon and Hollingshead, 2004; Hollingshead, 1998). Second, during the process of team reflexivity, team members overtly reflect upon and discuss about the team’s objectives, strategies, and processes. This allows the team to identify the current deficiencies at work (Wang et al., 2021), motivating members to share their expertise and knowledge to reconcile the discrepancies between the objectives and current performance. In this way, team reflexivity may eliminate their mutual boundaries and allow members to discover expertise within team. As each team member focuses on mutual area of expertise, they contribute to the overall efficiency of the TMS. Third, team reflexivity will enhance coordination within the team. By reflecting on their interactions and processes, team members can identify and address any gaps or overlaps in their collective knowledge. This leads to better coordination in information sharing and task execution, which is essential for the smooth operation of a TMS. In summary, team reflexivity plays a pivotal role in promoting the credibility, specialization, and coordination necessary for a well-functioning TMS.

As mentioned above, we conclude that team reflexivity will develop a trusting and collaborative environment among members, and also enable team members to clearly recognize the mutual expertise among the team, which benefits the formation of the main components of TMS including credibility, specialization, and coordination. Therefore, we propose the following hypothesis:

*H1:* Team reflexivity is positively related to TMS.

### 2.3 Team reflexivity and feedback-seeking behavior: the mediating role of TMS

Furthermore, we identify TMS as a critical determinant of team member’s feedback-seeking behavior. The exploration of the antecedents of feedback-seeking behavior has mostly been conducted from the perspective of the value-cost framework (Sung and Choi, 2021). When deciding whether or not to seek feedback from others, individuals will first assess the potential cost and value involved in the behavior. Specifically, the most obvious value is the possibility of obtaining diagnostic information helping to improve one’s performance and achieve the anticipated objectives (Ashford, 1986). In contrast, feedback-seeking behavior may also impose cost, including damage to one’s ego (such as receiving negative feedback) or one’s image (such as appearing unsure or incompetent) (Ashford, 1989). Thus, feedback-seeking behavior is more likely to emerge when the expected value exceeds its cost (Anseel et al., 2015).

As a critical factor, TMS can affect the perceived value and cost of feedback-seeking behavior in terms of credibility, specialization, and coordination. Firstly, when team members have a high level of credibility with the others of the team, they believe that the feedback they receive from others will be truthful and constructive, which will increase the perceived value of feedback-seeking behavior. At the same time, when team members have a high level of credibility with the others of the team, they are also less likely to fear embarrassment or humiliation, which are potential costs associated with feedback-seeking behavior. This increment in expected values and reduction in expected costs make the feedback-seeking behavior more appealing. Secondly, members’ perception of team specialization will increase the value of feedback-seeking behavior because they can receive input from those with a deep understanding of areas they may not be as familiar with. Specialized feedback is more likely to address specific issues and provide insights that lead to performance improvement (Ashford, 1986; Madison et al., 2021). Besides, members’ perception of team specialization can also mitigate the self-esteem damage that may come with receiving negative feedback. When team members recognize and respect each other’s areas of specialization, they are more likely to view negative feedback as a learning opportunity rather than a personal shortcoming, which will reduce the psychological cost of feedback-seeking behavior. Thirdly, the coordination established by TMS leads team members to believe that others will genuinely help each other to achieve the collective objectives, so they are convinced that it is reliable to seek feedback from others without being humiliated, which also increase the expected value and reduces the expected cost of feedback-seeking behavior.

In summary, TMS may increase the expected value and reduce the expected cost of feedback-seeking behavior, which encourages individuals to engage in this behavior more frequently. Therefore, we can propose the following hypothesis:

*H2:* TMS has a positive effect on team member’s feedback-seeking behavior.

To sum up, team reflexivity in which members overtly reflect upon and discuss the team’s objectives, strategies and processes may constitute the foundation for TMS structure of expertise allocation within a team. Thus, it enables members to understand each other’s expertise, enhances the credibility in their expertise, and facilitates a collaborative team atmosphere (Lewis, 2003), producing a favorable feedback environment. Therefore, based on transactive memory system theory, we contend that team reflexivity positively influences TMS, which in turn stimulates team member’s feedback-seeking behavior. Therefore, we propose the following hypothesis:

*H3:* TMS mediates the positive effect of team reflexivity on team member’s feedback-seeking behavior.

### 2.4 The moderating role of the SMMs

SMMs, characterized as the shared knowledge structure and cognition among team members concerning critical components of a team’s task environment (Lewis, 2003). In this study, we believe that SMMs will interact uniquely with team reflexivity to facilitate TMS. When team members engage in reflexivity, they are also essentially examining and adjusting their SMMs. A high level SMMs can make this process more effective, as it provides a clear framework for what needs to be examined and adjusted. Besides, researchers have provided evidence that TMS and SMMs are conceptually and empirically distinct constructs, and the efficient function of TMS relies on team members possessing a shared understanding of the cooperation patterns in the team (Austin, 2003; Lewis, 2003). Transactive memory system theory also indicates that team members with similar mental models are more likely to facilitate team TMS (Ellis, 2006; He and Hu, 2021). Therefore, we further suggest that the relationship of team reflexivity and TMS is moderated by SMMs.

As a system of knowledge structure and cognition collectively held by team members, SMMs contributes to the efficiency of team processes (Schmidtke and Cummings, 2017). On the one hand, an essential element of team reflexivity is the public discussion regarding team work. When SMMs are high, team members are more likely to reach consensus on team objectives and strategies, and the process of team reflexivity will be more effective (Wang et al., 2021), promoting cooperation and division among team members and further facilitating the formation of TMS. On the other hand, when team members hold a high level SMMs, they are more likely to accurately understand and reach consensus on team works, which in turn encourages members to coordinate their work smoothly and utilize each other’s expertise efficiently. Therefore, the respective expertise of team members can be effectively leveraged to improve the TMS.

In contrast, when the team is deficient in SMMs, it is problematic for members to reach a consensus on team tasks. Hence, during the team reflexivity process, the effectiveness of discussion may be compromised. On one hand, this will hinder members’ mutual understanding and credibility in others’ expertise; on the other hand, it will be inconducive to effectively integrate and utilize members’ knowledge and expertise, discouraging from obtaining the effective coordination of work. To sum up, we expect that SMMs would amplify the relationship between team reflexivity and TMS. Therefore, we posit the following hypothesis:

*H4:* SMMs moderates the positive relationship between team reflexivity and TMS, such that the strength of this relationship is more positive for teams with more similar SMMs.

## 3 Methodology

### 3.1 Sample and procedure

We recruited 280 employees and their direct leaders from 59 teams in different industries (e.g., service, manufacture, finance and IT) in China, and all of these teams are either management or R&D teams. We chose these samples for two reasons: Firstly, influenced by China’s collectivist culture, team reflexivity and feedback-seeking behavior are more likely to occur in the Chinese context. Secondly, team reflexivity is particularly important for both management teams and R&D teams. Furthermore, to improve data quality, the survey was anonymous and matched, each team leader and employee should fill out the identification number assigned to them correctly. Besides, in order to reduce possible common method bias, we collected the data in two waves with an interval of three months. At Time 1, questionnaires were sent to 280 employees from 59 teams. In specific, we invited employees to complete their demographic information, team reflexivity, and the SMMs. At the end, we screened the questionnaires and finally obtained valid questionnaires from 243 employees of 57 teams, with an effective response rate of 86.79%. At Time 2, three months later, we sent new questionnaires to 243 valid respondents, and asked them to complete a rating of the TMS. At the same time, we sent questionnaires to the direct 57 team leaders of 243 employees, which included their evaluation of employee’s feedback-seeking behavior. Ultimately, we received 213 valid questionnaires from 56 teams, with an effective response rate of 87.65%. In the final sample, 67.61% were male, 52.11% were below 25 years old; About 37.56% had been working for their companies between one and three years, and 87.79% had bachelor’s degrees or higher.

### 3.2 Measures

Considering the measurement of variables, we used scales that have been shown to have high reliability and validity to measure our target variables. A five-point Likert scale (1 = “strongly disagree,” 5 = “strongly agree”) was used in the survey.

#### 3.2.1 Team reflexivity

We measured team reflexivity using the 9-item scale adapted from Tjosvold et al. (2004) and Carter and West (1998). Sample items included “We regularly discuss whether the team is working effectively together.” The Cronbach’s alpha of this measurement instrument was 0.915.

#### 3.2.2 Transactive memory system

We measured TMS using the 15-item scale adapted from Lewis (2003). Sample items included “Different team members are responsible for expertise in different areas.” The Cronbach’s alpha of this measurement instrument was 0.927.

#### 3.2.3 Shared mental models

We measured SMMs using the 6-item scale from Cannon-Bowers et al. (1990). Sample items included “Team members are familiar with the skills and competencies of other members.” The Cronbach’s alpha of this measurement instrument was 0.870.

#### 3.2.4 Feedback-seeking behavior

The 11-item scale adapted from Callister et al. (1999) was used to measure employees’ feedback-seeking behavior, which is rated by the direct leaders of the participants. Sample items for employees’ feedback-seeking behavior included “He/She asks me if he/she meets job requirements.” The Cronbach’s alpha of this measurement instrument was 0.917.

#### 3.2.5 Control variables

According to existing research on individual feedback-seeking behavior (Ferris et al., 2015), we controlled for factors that may have influenced this research, such as employees’ age, gender, education level, and tenure in the current company. Besides, the participants come from different teams and the data is nested, so we further control for team size in the multi-level analysis.

### 3.3 Data aggregation

Our research model presents the effect of team reflexivity on team member’s feedback-seeking behavior with a multilevel nature and the employee data we collected were nested within the team. At the same time, according to the definition of team reflexivity, TMS, and SMMs, we can see that these variables represent the collective nature of the team and only make sense when aggregated to the team level. So, we further analyzed whether the data could be aggregated. Firstly, we performed a one-way random-effects ANOVA and calculated the ICC values for the team-level focal variables. The ICC(1) values of team reflexivity, TMS, and SMMs were 0.37, 0.22, and 0.47, respectively; while the ICC(2) values of these constructs were 0.69, 0.51, and 0.77, respectively. The results indicated that team reflexivity, TMS, and SMMs varied across teams. Secondly, we further calculated the reliability of score within group (R_wg_) across teams to test the within-team agreement (James et al., 1984). The R_wg_ values of team reflexivity, TMS, and SMMs were 0.943, 0.966, and 0.937, respectively, demonstrating a high level of agreement within the teams. Taken together, the results showed that the target variables (team reflexivity, TMS, and SMMs) can be appropriately aggregated to the team level.

## 4 Results

### 4.1 Confirmatory factor analyses

Firstly, the discriminant validity of the focal variables was tested by a series of CFA analyses using Mplus 7.4 software before we tested the hypotheses. The results are shown in Table 1, the hypothesized four-factor model (M0), which included team reflexivity, TMS, SMMs and feedback-seeking behavior fits to the data well (*χ*^2^ = 317.706, df = 224, CFI = 0.938, TLI = 0.929, RMSEA = 0.044, SRMR = 0.051). Besides, the results also showed that the four-factor model had the best fit validity compared to the other models, which included all three-factor models (any two of the four factors were combined). Therefore, the findings suggested that our target variables are distinctive from each other and the distinctiveness of them was supported.

**Table 1:** Results of the confirmatory factor analysis.

Model	*χ* ^2^	df	CFI	TLI	RMSEA	SRMR
Four-factor model (TR, TMS, SMMs, FSB)	317.706	224	0.938	0.929	0.044	0.051
Three-factor model (TR + TMS, SMMs, FSB)	454.214	227	0.849	0.831	0.069	0.063
Three-factor model (TR + SMMs, TMS, FSB)	442.683	227	0.856	0.840	0.067	0.065
Three-factor model (TR + FSB, TMS, SMMs)	378.129	227	0.899	0.888	0.056	0.061
Three-factor model (TR, TMS + SMMs, FSB)	425.726	227	0.868	0.852	0.064	0.061
Three-factor model (TR, TMS + FSB, SMMs)	364.553	227	0.908	0.898	0.053	0.057
Three-factor model (TR, TMS, SMMs + FSB)	362.109	227	0.910	0.900	0.053	0.056

*N = 213. TR, team reflexivity; TMS, transactive memory system; SMMs, shared mental models; FSB, feedback-seeking behavior.*

### 4.2 Hypotheses testing

The data of this study contained both individual and team level, with employees being nested within their teams. The descriptive statistics and correlations of the variables are shown in Table 2. Then, we further performed a multi-level analysis using Mplus7.4 software to test our hypotheses. The result of coefficient estimates for our hypotheses are shown in Table 3. To test Hypothesis 1, we regressed the TMS on team reflexivity and the results demonstrated that team reflexivity was positively related to TMS (*β* = 0.388, *p* < 0.01). This means that team reflexivity will change in the same direction as TMS, that is, when the level of team reflexivity is high, the TMS will also be higher. Therefore, the Hypothesis 1 was supported.

**Table 2:** Descriptive statistics.

Variable	Mean	SD	1	2	3	4	5	6	7	8	9
1. Team size	3.99	0.849	—	—	—	—	—	−0.039	−0.165^*^	0.018	—
2. Employee gender	1.320	0.469	—	—	—	—	—	—	—	—	—
3. Employee age	1.460	0.536	—	−0.039	—	—	—	—	—	—	—
4. Employee education	1.990	0.523	—	−0.161^*^	−0.052	—	—	—	—	—	—
5. Employee tenure	1.460	0.578	—	−0.025	0.136^*^	−0.017	—	—	—	—	—
6. TR	4.015	0.533	—	−0.136^*^	−0.138^*^	−0.031	−0.024	—	0.524^**^	0.610^**^	—
7. SMMs	3.822	0.590	—	−0.109	−0.136^*^	0.040	−0.029	0.487^**^	—	0.661^**^	—
8. TMS	4.124	0.564	—	0.005	−0.169^*^	0.074	0.005	0.564^**^	0.560^**^	—	—
9. FSB	3.322	0.402	—	0.209^**^	−0.059	0.018	0.036	0.212^**^	0.276^**^	0.289^**^	—

*N* = 213. *p < 0.05; **p < 0.01. Correlations below the diagonal represent individual level correlations, and correlations above the diagonal represent team level correlations.

**Table 3:** Results of hypothesis testing.

	TMS			Feedback-seeking behavior		
	Estimate	S.E.	95% CI	Estimate	S.E.	95% CI
*Within level*						
Employee gender				0.090^**^	0.031	[0.028, 0.152]
Employee age				0.058^*^	0.025	[0.009, 0.107]
Employee education				−0.025	0.026	[−0.076, 0.026]
Employee tenure				0.000	0.031	[−0.061, 0.061]
*Between level*						
Team size	0.051	0.034	[−0.017, 0.118]			
Team reflexivity	0.388^**^	0.094	[0.204, 0.572]	0.072	0.140	[−0.202, 0.346]
TMS				0.488^**^	0.128	[0.238, 0.738]
SMMs	0.367^**^	0.059	[0.252, 0.482]			
Team reflexivity × SMMs	0.409^*^	0.208	[0.002, 0.816]			

*N* = 213, Team = 56. *p < 0.05; **p < 0.01.

Further more, to test Hypothesis 2, team member’s feedback-seeking behavior was regressed on TMS and control variables. Results showed that TMS was positively related to team member’s feedback-seeking behavior (*β* = 0.488, *p* < 0.01). This means that TMS will change in the same direction as team member’s feedback-seeking behavior, that is, when the level of TMS is high, team members will engage in more feedback-seeking behaviors. In addition, there was a positive indirect effect of team reflexivity on team member’s feedback-seeking behavior via TMS (*β* = 0.189) and the 95% confidence interval is [0.053, 0.325], excluding zero (see Table 4). Therefore, Hypothesis 2 and Hypothesis 3 were supported.

**Table 4:** Results of mediating effect.

Path	Estimate	S.E.	95% CI	Hypothesis test
Team reflexivity→TMS→ Feedback-seeking behavior	0.189^**^	0.069	[0.053, 0.325]	Support H3

*N* = 213, Team = 56. *p < 0.05; **p < 0.01.

**Figure 2:**
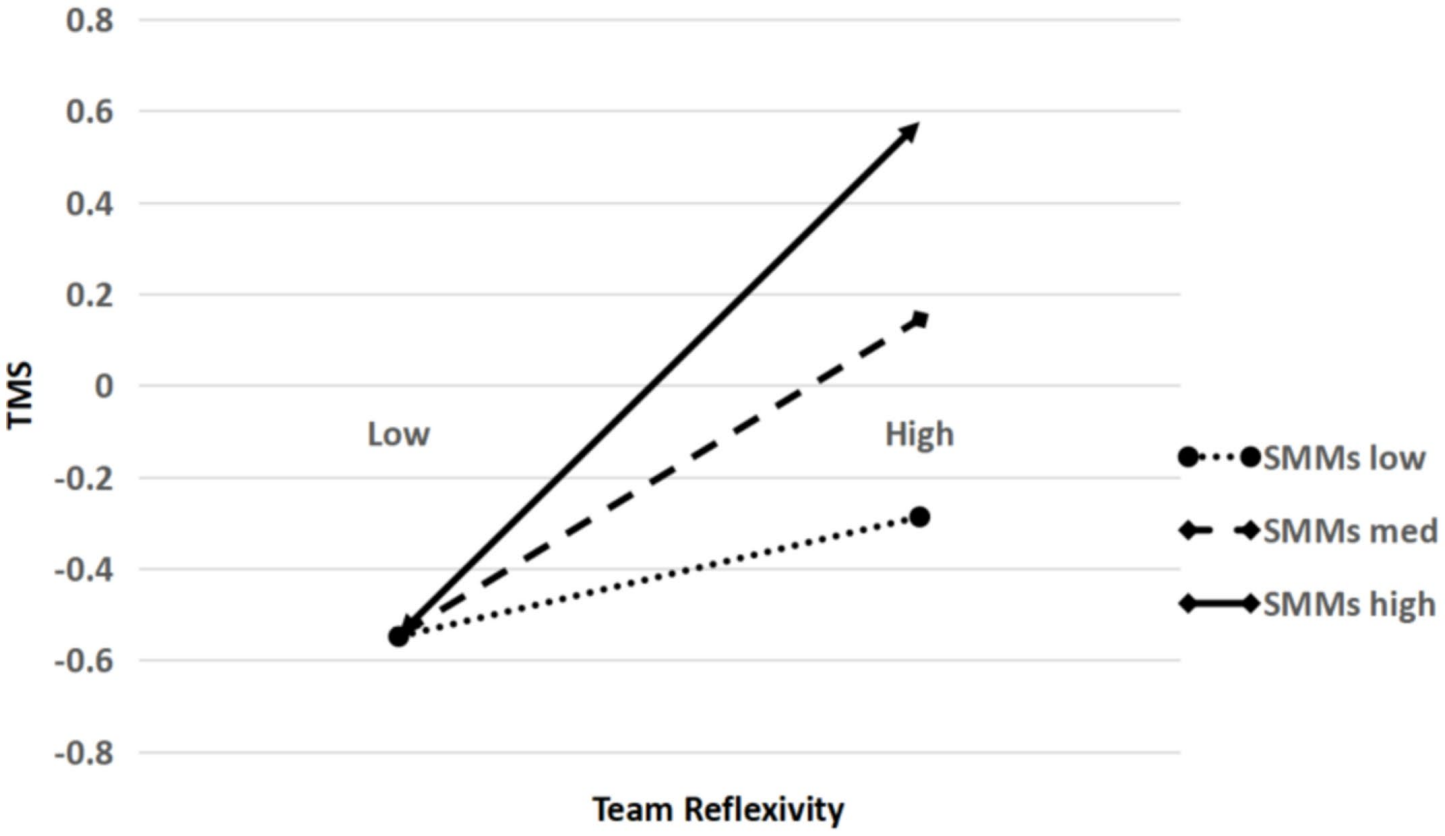
Moderating effect of shared mental models.

Lastly, we centered team reflexivity and SMMs and then interacted them to further test the moderating effect. The analysis results (see Table 3) showed that the interaction effect between team reflexivity and SMMs on TMS was significant (*β* = 0.409, *p* < 0.05). Moreover, following the suggestion of Aiken et al. (1990), we further examined the significance of simple slopes at different levels of SMMs (1 SD above the mean value and 1 SD below the mean value). Figure 2 and Table 5 showed the result of simple slope testing. In particular, the relationship between team reflexivity and TMS was stronger when SMMs was higher (*β* = 0.572, *p* < 0.01) but weaker when SMMs was lower (*β* = 0.204, *p* < 0.05); the difference between these two conditions (1 SD above the mean value and 1 SD below the mean value) was significant (*β* = 0.368, *p* < 0.05). Taken together, Hypothesis 4 was supported.

**Table 5:** Results of simple slope analyses.

SMMs	Estimate of simple slope	S.E.	95% CI	Hypothesis test
High (+SD)	0.572^**^	0.167	[0.245, 0.898]	Support H4
Low (-SD)	0.204^*^	0.085	[0.037, 0.371]	
Difference	0.368^*^	0.187	[0.002, 0.734]	

*N* = 213, Team = 56. Simple slope indicates the relationship between team reflexivity and transactive memory system. *p < 0.05; **p < 0.01.

## 5 Discussion

Considering the importance of member’s feedback-seeking behavior in teams, it is critical to explore which team factors influence team member’s feedback-seeking behavior. Based on transactive memory system theory, our study responded to this question and proposed that team reflexivity is an important predictor of team member’s feedback-seeking behavior and subsequently proposed four hypotheses. The results of the hypotheses test in the research are summarized in Table 6. Specifically, team reflexivity is positively related to TMS, and TMS has a positive effect on team member’s feedback-seeking behavior. In addition, team reflexivity indirectly affected member’s feedback-seeking behavior through TMS and that SMMs moderated the effect of team reflexivity on TMS, such that these effects would be stronger when the team with a high-level SMMs. Overall, this study has several theoretical and practical implications.

**Table 6:** Summary of the hypotheses test.

Hypotheses	Content of the hypotheses	Test results
Hypothesis 1	Team reflexivity is positively related to TMS.	Support
Hypothesis 2	TMS has a positive effect on team member’s feedback-seeking behavior.	Support
Hypothesis 3	TMS mediates the positive effect of team reflexivity on team member’s feedback-seeking behavior.	Support
Hypothesis 4	SMMs moderates the positive relationship between team reflexivity and TMS, such that the strength of this relationship is more positive for teams with more similar SMMs.	Support

### 5.1 Theoretical implications

This study contributes to the current literature in three aspects. Firstly, this study advances the literature on the outcomes of team reflexivity at the individual level by proposing and examining a multilevel model. While the extant research on exploring team reflexivity outcomes are mostly focused on the teams (Yang et al., 2020; Wu et al., 2019; Schmutz et al., 2018; Schippers et al., 2012). Only a few studies have examined the impact of team reflexivity on team members (Chen et al., 2018; Wang et al., 2021; Wang et al., 2022). In addition, previous research also indicated that team reflexivity may produce a supportive feedback environment (Sung et al., 2019; Wu et al., 2014), but they did not further test the relationship. To advance the literature on team reflexivity and refine existing research findings, this study focused on the individual outcome, proposed and examined the multilevel effect of team reflexivity on team member’s feedback-seeking behavior.

Secondly, this research provided a new theoretical insight on the mechanisms by which team reflexivity affects individual’s behavior in the perspective of team’s cognitive process. Existing studies have pointed out that job demands, control, support (Chen et al., 2018), knowledge sharing (Wang et al., 2021) and individual intellectual capital (Wang et al., 2022) mediate the effect of team reflexivity on team members. These research mainly focused on the mechanisms of individual factors, may overlook that the impact of team reflexivity on employees may not be straightforward, as some other team factors could potentially play a pivotal role in this regard. Thus, based on transactive memory system theory, we proposed and examined TMS as a mediator in the relationship between team reflexivity and team member’s feedback-seeking behavior, providing a new perspective (team cognitive process) on the mechanism of team reflexivity influencing member’s behavior. Additionally, this study enriched trasactive memory system theory by explain the relationship between team reflexivity and individual behaviors. Existing studies explored the mediating effect of TMS in the relationship between several types of antecedents (e.g. management practices, team characteristics and environment characteristics) and performance outcomes (e.g., Chiang et al., 2014; Argote et al., 2018; Bachrach et al., 2018; Huang and Chen, 2018). Thus, our study provided a new application of trasactive memory system theory by explain the relationship between the team interaction characteristic and the individual-behavior outcome.

Thirdly, our study further makes a contribution to team reflexivity literature by exploring an essential boundary condition for its effects on TMS. Existing studies have mainly investigated the moderating effects in the process of team reflexivity, such as team tenure (Chen et al., 2018), team size (Schmutz et al., 2018), team diversity (Yang et al., 2020), team context (Schippers et al., 2012) and leadership style (Wang et al., 2021), etc. However, coherent cognition among team members would improve the effectiveness of team reflexivity (Konradt et al., 2021), which has not attracted the attention of scholars. Therefore, according to transactive memory system theory, we examined the moderating role of SMMs featuring team coherent cognition in the effect of team reflexivity on TMS and team member’s feedback-seeking behavior. By doing so, we enriched the literature of team reflexivity and provided a fuller understanding of the effects of team reflexivity on individual-level outcomes.

### 5.2 Practical implications

Practically, the present study also offers several valuable implications. Firstly, the results encourage team leaders to recognize the importance of team reflexivity and better leverage team reflexivity to enhance team member’s feedback-seeking behavior. Specifically, in the perspective of team routine, team leaders should actively create an atmosphere of adequate communication and mutual trust among team members, and encourage them to discuss team objectives and processes in public, thereby increasing the expected value and reducing the cost of feedback-seeking behavior. However, there are limits to what leaders can do individually, and organizations need to find more ways to support the team reflexivity in the management process, for example, setting up guided reflexivity sessions, in other words, team debriefings or after action reviews (Leblanc et al., 2022). These ways have been proved to be efficient to encourage reflexive activities of employees in following work routine (Konradt et al., 2015).

Second, the findings also show that team reflexivity can facilitate team member’s feedback-seeking behavior by enhancing TMS. Therefore, leaders and organizations should focus more on the development of the TMS. Specifically, in the process of recruiting, the leader and human resource department should have regard to building a well-conceived knowledge structure by incorporating individuals with differentiated and complementary expertise. Meanwhile, during team operations, the team should be expected to provide platforms and opportunities conducive to communication among members. Additionally, organizations should enhance the exchange of knowledge, information and emotions among members to accelerate the flow of knowledge and information. At the same time, organizations could set team communication space and fixed communication schedule in order to build a foundation of practice. By doing so, it will foster mutual understanding and trust, and strengthen TMS of the team, thus encouraging individuals to engage in feedback-seeking behavior more frequently.

Third, our study reveals that SMMs, as a key moderator, amplifies the positive effects of team reflexivity on TMS in teams. Accordingly, managers should keep track of the development of SMMs by encouraging team members to communicate information and share knowledge for achieving the common objectives, which may promote members to generate a consistent expectation for objectives and a collective understanding of task-related knowledge. In the perspective of human resource management, organizations can implement training on interpersonal communication and interaction to foster a common understand about social roles and norms, which can help to built SMMs of teams (Arendt et al., 2024). As such, SMMs can effectively improve team communication, trust and collaboration, leveraging upon member’s knowledge and expertise.

### 5.3 Limitations and future research directions

Although this study has some theoretical and practical implications, there are still some limitations that can be addressed in the future. First, based on transactive memory system theory, we proposed and examined TMS and SMMs as the mediator and the moderator in the relationship of team reflexivity-team member’s feedback-seeking behavior. Yet, there may be other theoretical perspectives that can explain the relationship between team reflexivity and team member’s feedback-seeking behavior, such as social cognitive theory, self-determination theory, etc. At the same time, there may be other factors that may mediate the relationship between team reflexivity and team member’s feedback-seeking behavior, including individual-level factor (e.g., the motivation of feedback-seeking behavior, self-efficacy), internal team-level factors (e.g., inter-member trust, conflict resolution), etc. Thus, in the future, researchers can further explore other significant mechanisms how team reflexivity affects team members from different perspectives, and deeply discuss interplay between TMS, SMMs and other internal team-level factors.

Second, given the availability of data, we only conducted the study with companies in different regions of China. However, as Chinese employees may be profoundly influenced by China’s collectivist culture, their acceptance of feedback-seeking behavior may differ from that in other countries. Therefore, we need to be cautious when generalizing our conclusions to other countries. At the same time, we also call for more studies to increase the sample size and include a large number of companies from other countries to enhance the generalizability of the findings in the future.

Third, the measure of team reflexivity in our research focused mainly on the quantity and number of reflection. However, several scholars have pointed out that assessing quality (i.e., the depth of information processing) in team reflexivity constructs could be more relevant to team and individual than assessing quantity (Moreland and McMinn, 2010; Schippers et al., 2014; Otte et al., 2017). Therefore, the present study did not consider comprehensively when exploring the mechanism of action of team reflexivity, and in the future, the quality and quantity of team reflexivity should be both taken into account to reveal its unique mechanism.

Fourth, according to existing research on individual feedback-seeking behavior (Ferris et al., 2015), we controlled for factors that may have influenced this research, such as employees’ age, gender, education level, and tenure in the current company. However, we ignored some other team factors, such as industry type and team tenure, which may also influence team reflexivity, TMS and SMMs in this study. Therefore, we will fully consider the possible influencing factors of each variable and reasonably select control variables to ensure the accuracy of the research results in future related studies.

Fifth, although we used a two-wave, time-lagged design to minimize common method bias, we measure each construct only once, and since this study was essentially cross-sectional, causal inferences could not be established explicitly. The results reported in the text can only represent the correlation between the variables, and cannot accurately reflect the causal inference. Therefore, longitudinal or experimental studies could be conducted in the future to better validate the causal inferences of research model in this paper.

## Data Availability

The original contributions presented in the study are included in the article/supplementary material, further inquiries can be directed to the corresponding author/s.
